# Supplementary material for: Genetic determinants underlying the progressive phenotype of β-lactam/β-lactamase inhibitor resistance in Escherichia coli
Source: Microbiol Spectr. 2023 Oct 6;11(6):e02221-23. doi: 10.1128/spectrum.02221-23 (PMC10715226; doi:10.1128/spectrum.02221-23)
Supplement: Supplemental figures — Fig. S1 and S2. [file spectrum.02221-23-s0001.docx]

**Supplemental Figures**

 **FIG S1. Study Population and BL/BLI MIC Distribution Across Each Non-Susceptible Group.** (A) Overview of total *E. coli* bacteremia isolates detected from May 1^st^, 2015, to April 30^th^, 2020, with their respective ESRI phenotype groups. S = susceptible, NS = non-susceptible. (B) Categorical distribution of minimum inhibitory concentration (MIC; μg/mL) values across each respective BL/BLI non-susceptibility group designation for AMP, SAM, AMC, and TZP respectively. S = susceptible, I = intermediate, and R = resistant according to CLSI guidelines. Intermediate isolates are shaded gray.


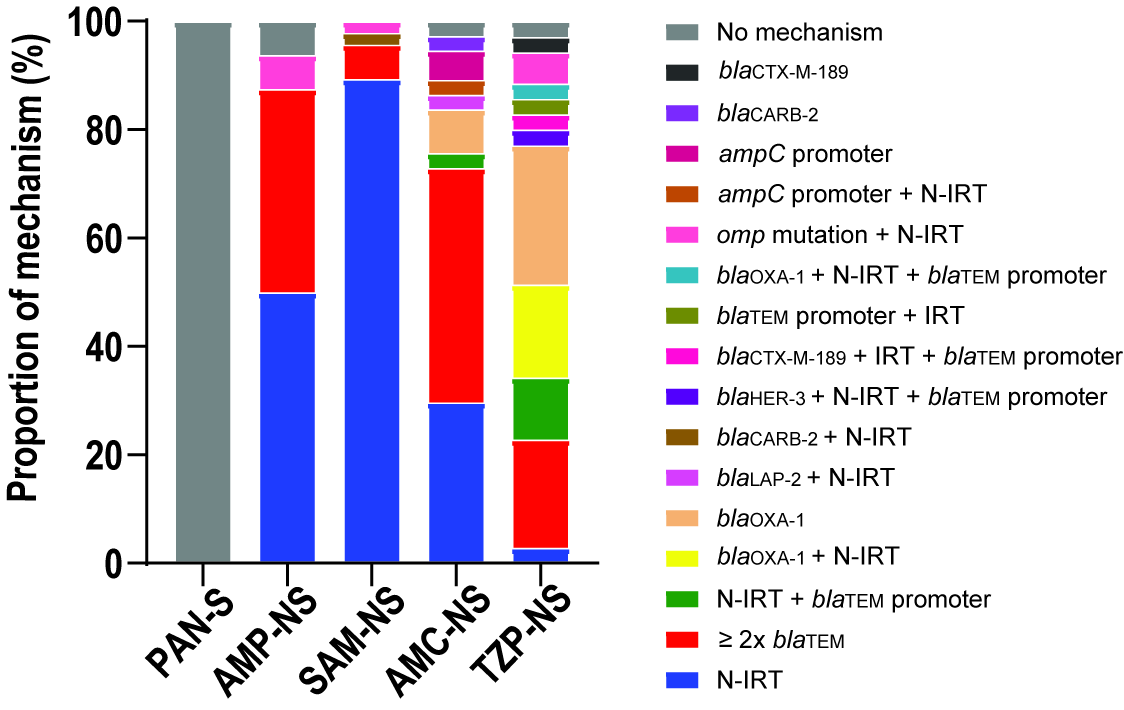


**FIG S2. Proportion of BL/BLI genetic mechanisms detected across *E. coli* isolates by BL/BLI phenotype.** Each of the respective combinations of genetic mechanisms are listed across each of the BL/BLI phenotypes and labelled accordingly in the legend. N-IRT = non-inhibitor resistant *bla*_TEM_ variant (usually *bla*_TEM-1_). IRT = inhibitor resistant *bla*_TEM_ variant, promoter = promoter variant leading to increased gene expression, ≥ 2x *bla*_TEM_ = copy number variation ≥ 2.
